# Diazotrophic *Azotobacter salinestris* YRNF3: a probable calcite-solubilizing bio-agent for improving the calcareous soil properties

**DOI:** 10.1038/s41598-023-47924-w

**Published:** 2023-11-23

**Authors:** Younes M Rashad, Mohamed Hafez, Mohamed Rashad

**Affiliations:** 1 Plant Protection and Biomolecular Diagnosis Department, Arid Lands Cultivation Research Institute (ALCRI), City of Scientific Research and Technological Applications (SRTA-City), New Borg El-Arab, 21934 Egypt; 2 Land and Water Technologies Department, Arid Lands Cultivation Research Institute (ALCRI), City of Scientific Research and Technological Applications (SRTA-City), New Borg El-Arab, 21934 Egypt

**Keywords:** Element cycles, Agroecology

## Abstract

Calcareous soils are characterized by a high calcium carbonate content (calcite), which plays a crucial role in the soil structure, plant growth, and nutrient availability. The high content of CaCO_3_ leads to the increment of the soil alkalinity, which results in a lowering of the nutrient availability causing a challenge for the agriculture in these soils. In this study, the calcite-solubilizing potential of the diazotrophic *Azotobacter salinestris* YRNF3 was investigated in vitro as a probable bio-agent for enhancing the calcareous soils properties such as soil pH and nutrient availability. Twelve diazotrophic bacterial strains were isolated from wheat rhizosphere collected from different wheat-cultivated fields in five Egyptian governorates. Using Nessler’s reagent, all isolated bacterial strains were found to have the ability to produce ammonia. By amplification of *nifH* gene, a PCR product of 450 bp was obtained for all isolated bacterial strains. For each isolate, three biological and three technical replicates were applied. All isolated diazotrophic bacteria were qualitatively screened for their calcite-solubilizing ability. To quantitatively investigate the calcite-solubilizing potential of *A. salinestris* YRNF3 in vitro, changes in the contents of soluble calcium (Ca^2+^), bicarbonate (HCO_3_^−^), total nitrogen (TN), total protein (TP), and pH were daily measured in its culture filtrate along 10 days of incubation. The results showed that the pH values in the culture filtrate ranged from 5.73 to 7.32. Concentration of Ca^2+^ and HCO_3_^−^ in the culture filtrate significantly decreased with the increment in the incubation time, while concentration of TN increased along the time. The highest TN concentration (0.0807 gL^−1^) was observed on days 4 and 5, compared to that of the day 0 (0.0014 gL^−1^). Content of TP in the culture filtrate also significantly increased along the incubation period. The highest TP content was recorded in day 4 (0.0505%), while no TP content was recorded on day 0. Furthermore, data obtained revealed that *A. salinestris* YRNF3 produced acid phosphatase at low activity (5.4 U mL^−1^). HPLC analysis of the culture filtrate indicated production of different organic acids, namely lactic acid (82.57 mg mL^−1^), formic acid (46.8 mg mL^−1^), while acetic acid was detected in a low quantity (3.11 mg mL^−1^). For each analysis, three replicates of each treatment were analyzed. Means of the tested treatments were compared using Tukey's HSD test at *p* ≤ 0.05. In conclusion, findings of this work suggested that *A. salinestris* YRNF3 has the potential to be a probable bioagent to be used for the reclamation of the calcareous soils by solubilizing CaCO_3_, improving soil fertility, and promoting plant growth. However, further studies are needed to investigate its field application and their long-term effects on the soil properties and plant productivity. To the best of the author's knowledge, this is the first study reporting the calcite-solubilizing ability of a nitrogen-fixing bacteria. Having these two abilities by one microorganism is a unique feature, which qualifies it as a promising bioagent for reclamation of the calcareous soils.

## Introduction

Calcareous soils are widespread in arid and semiarid regions, covering about 30% of the world's land area, including Egypt^1^. These soils have a high content of calcium carbonate (CaCO_3_), which makes them highly alkaline and reduces the nutrient availability for plants. Calcite solubilization is a natural process that occurs in soil environments, where microorganisms can dissolve the calcite leading to the release of calcium and carbonate ions^2^. Microbial calcite solubilization can decrease soil pH releasing the nutrients from the soil matrix and making them available for the plant uptake^3^.

Various methods have been used for soil reclamation, including chemical and physical methods. Chemical methods such as sulfuric acid and gypsum application have been used to reduce the soil pH and improve the nutrient availability^4^. However, these methods have drawbacks such as the high cost, negative environmental impacts, and long-term effects on the soil properties. Physical methods such as the deep plowing and subsoiling have been used to improve soil structure and reduce soil alkalinity^5^. However, these methods may cause soil erosion and compaction^6^.

Microbial calcite solubilization is a potential, eco-friendly, and sustainable alternative to the chemical and physical methods, as it is a natural process that can improve the soil properties without any negative effects. Several studies have investigated the ability of microorganisms to precipitate calcite for soil stabilization and improving soil strength and stiffness^7–9^. However, reports dealing with microbial calcite solubilization are limited. In this regard, Tamilselvi et al.,^10^ reported the calcite-solubilizaing ability of *Brevibacterium* sp. SOTI06 released 18.6% of the native CaCO_3_ solution. In another study, Peper et al.^3^ isolated 65 calcite-solubilizing bacterial (CSB) isolates belonging to 10 genera from a peanut pegging zone in Georgia. The main responsible mode of action for calcite-solubilization is the production of organic acids such as citric, gluconic, and acetic acids^10^. In addition, the production of sanazine pigment was also reported^11^.

The use of microorganisms to enhance the soil fertility and plant growth has gained attention in the recent years due to their potential to provide sustainable solutions to the agricultural challenges^12–15^. Diazotrophic bacteria are capable to fix the atmospheric nitrogen to the inorganic form (NH_4_^+^) which is available to the plant via the nitrogenase enzyme^16^. Members of various bacterial genera, known as nitrogen fixers, are categorized into two types. The first type is the symbiotic bacteria which live in a mutualistic relationship with specific plants such as *Rhizobium* spp. that live in association with the leguminous plants^17^. The second type is the free-living (asymbiotic) bacteria such as *Azotobacter* spp.*, Beijerinckia* spp. and cyanobacteria^18^. Furthermore, nitrogen fixing bacteria are known to have another growth promoting effects on the plant via production of phytohormones, signaling molecules, and siderophores, trigging plant tolerance to the biotic and abiotic stresses, as well as minerals solubilization^19–21^. Members of the genus *Azotobacter* are the most prevalent asymbiotic nitrogen-fixing and plant growth-promoting bacteria^22^. Among them, *A. salinestris* is known to pose a high potential nitrogen-fixing ability, tolerance to salinity stress up to 8% NaCl and the common pesticides, and an efficient antifungal potential^23^. As an initial phase within a long-term project aimed at the development of a multifunctional bio-product for the reclamation of calcareous soils and the enhancement of the soil fertility, this study had the following objectives: (1) to isolate a variety of free-living nitrogen-fixing bacteria from diverse soil samples, (2) to assess their calcite-solubilization activity, (3) to identify the most efficient isolate, (4) to estimate its in vitro calcite-solubilization potential, and (5) to investigate the proposed mode of action.. 

## Results and discussion

### Isolation of the diazotrophic bacteria

In this study, twenty-five soil samples from different agricultural fields located at five Egyptian governorates representing varied climatic conditions and agricultural practices and soil types were collected. Twelve bacterial isolates, with varied colony shape and color, were isolated from the collected samples using the nitrogen-free (NF) medium. In this regard, the highest number of isolated bacteria was recorded in the New Valley and Al-Behera governorates (4 bacterial strains), followed by Alexandria and Sinai governorates (2 bacterial strains), while no bacterial strains were isolated from the soil samples from Port Said governorate.

**Figure 1:**
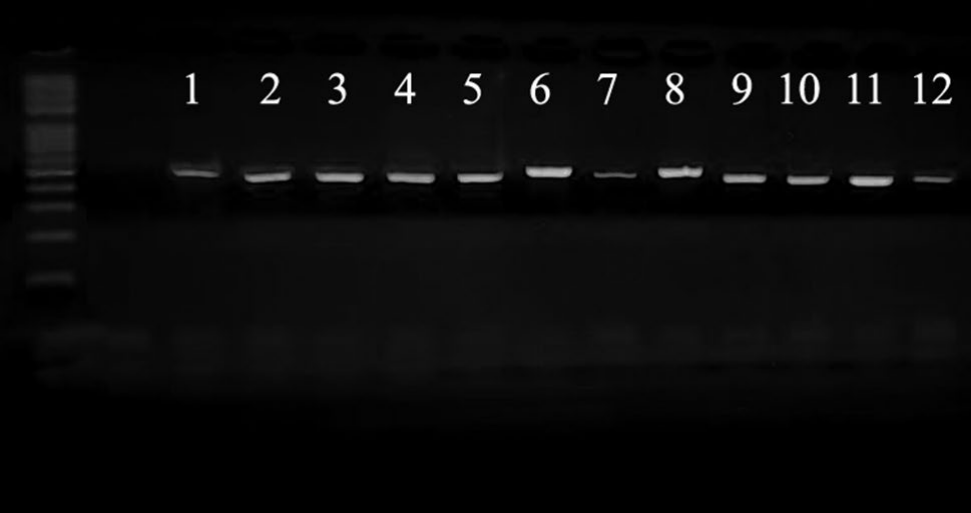
Agarose gel electrophoresis of the PCR product of the nitrogenase gene (*nifH*) in the isolated diazotrophic bacterial strains, where 1: YRNF1, 2: YRNF2, 3: YRNF3, 4: YRNF4, 5: YRNF5, 6: YRNF6, 7: YRNF7, 8: YRNF8, 9: YRNF9, 10: YRNF10, 11: YRNF11, and 12: YRNF12.

Using the Nessler’s reagent, all isolated bacterial strains were found to be positive for ammonia production which may support their ability to fix the atmospheric nitrogen. To confirm the diazotrophic property, all isolated bacterial strains were subjected to amplification of *nifH* gene using PCR, which is universally used as a biomarker for the nitrogen-fixation ability. In this concern, a PCR product of 450 bp was obtained for all isolated bacterial strains confirming their diazotrophic potential. The PCR-amplified products of *nifH* gene of all isolated bacterial strains are shown in Fig. 1.

Diazotrophic microorganisms are globally significant because they are the only biological source of the fixed nitrogen in their ecosystems. Nitrogen fixation is an enzymatic process which has been carried out via the nitrogenase reductase, coded by *nifH* gene, by which the atmospheric nitrogen (N_2_) transforms to ammonium equivalents that are available to the plants^24^. *nifH* has become the marker gene of choice for studying biodiversity, distribution, ecology and phylogeny of the diazotrophic organisms^25^.

According to Delgado-Baquerizo et al.^26^, soil pH significantly affects abundance and diversity of their content of bacterial communities. However, diazotrophic bacteria tend to be affected by other physicochemical properties of the soil. For example, nitrogen fertilization suppresses their abundance and taxon composition, while potassium and phosphorus fertilization enhances it^27^. Song et al.^28^ found that the community structure of the nitrogen-fixing bacteria considerably varied based on the moisture, pH, salinity, contents of nitrogen, carbon, and sulphur. Severin et al. reported the negative effect of the high salinity on the nitrogenase activity and *nifH* expression in *Cyanobacteria*^28^, while other diazotrophs were found to inhabit the sea and ocean water^29^.

### Screening the isolated bacterial strains for their calcite-solubilization ability

**Figure 2:**
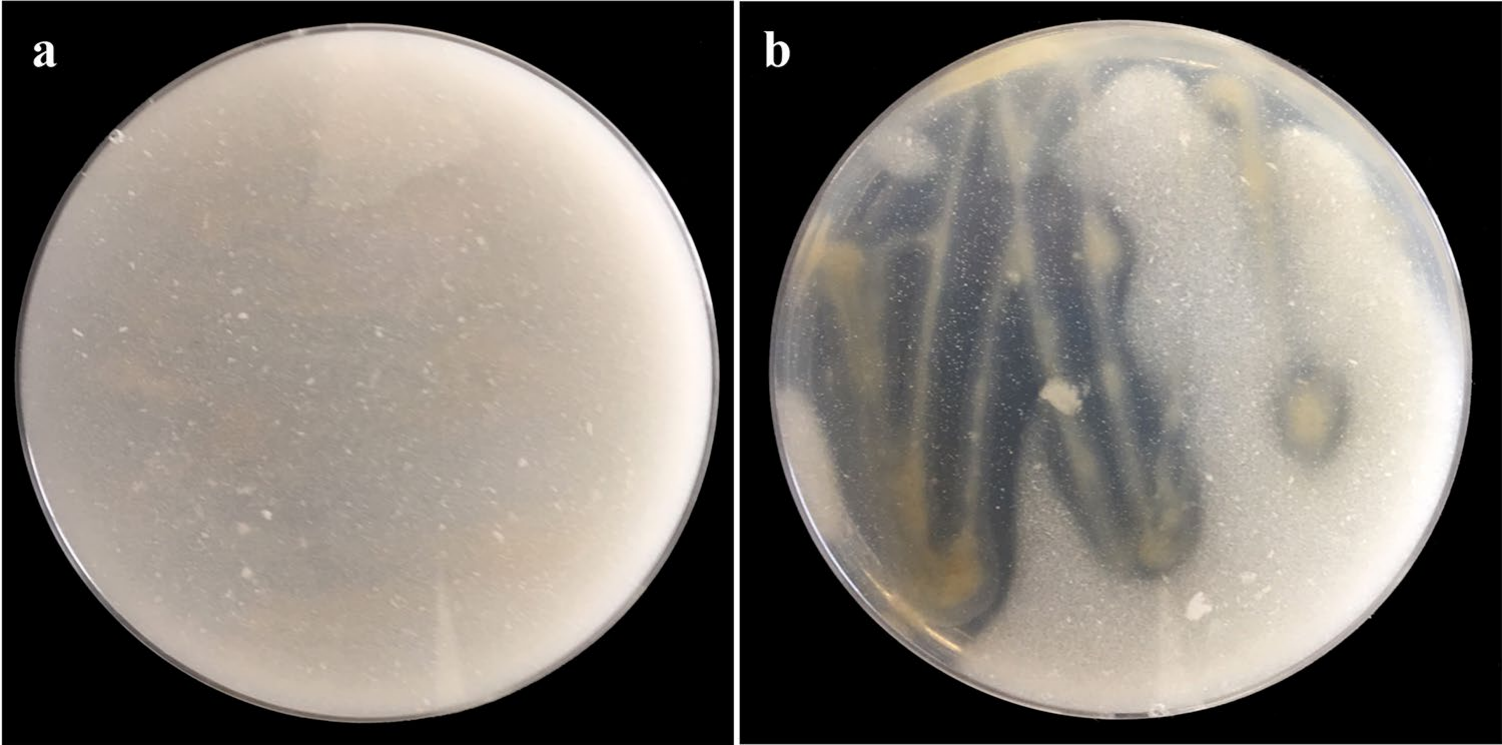
A photograph shows a clear zone formation by the diazotrophic bacterial strain YRNF3 indicating its calcite-solubilization potential, where (**a**) control Devenze-Bruni agar (DBA) plate, and (**b**) inoculated plate.

All isolated diazotrophic bacteria were qualitatively screened for their calcite-solubilization ability. Results obtained revealed that the isolated diazotrophic bacterial strains showed medium to low solubilization index values (0.41–3.04), except one strain (YRNF3) which showed a maximum solubilization index value 5.9 (Table 1). This strain was selected for the next evaluation tests. Calcite-solubilization potential of the diazotrophic bacterial strain YRNF3 is illustrated in Fig. 2. In general, the diazotrophic bacteria differ in their bioactivities and abilities, due to their high genetic diversity, which qualify them to adapt with different types of ecosystems. In addition to the nitrogen fixation, some diazotrophic bacteria have the ability to dissolute the rock phosphates^29^. Assistance in phytoremediation of heavy metals has been also reported for the diazotrophic bacteria^30^. On the contrary, to the best of the author's knowledge, no studies have been conducted on diazotrophic bacteria with a calcite-solubilization ability. Therefore, this study was aimed to isolate an efficient diazotrophic bacteria with a calcite-solubilization ability.

**Table 1:** Solubilization index for the isolated diazotrophic bacteria.

Strain code	Solubilization index	Strain code	Solubilization index
YRNF1	0.83	YRNF7	1.14
YRNF2	2.24	YRNF8	2.20
YRNF3	5.90	YRNF9	0.75
YRNF4	2.55	YRNF10	1.77
YRNF5	1.91	YRNF11	0.93
YRNF6	3.04	YRNF12	0.41

### Molecular identification of the diazotrophic bacteria YRNF3

**Figure 3:**
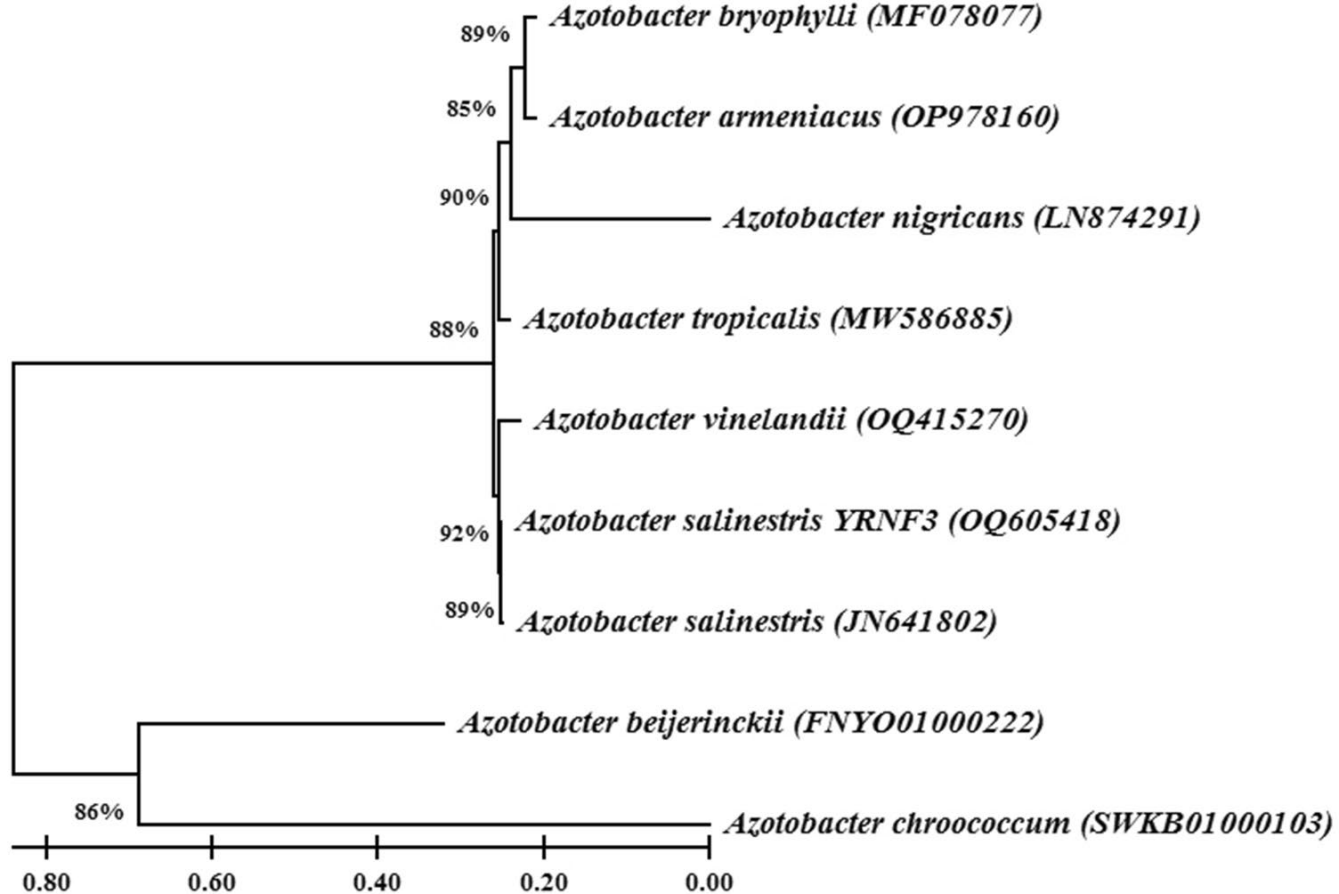
A Phylogenetic tree indicates the relationship between the diazotrophic bacteria *Azotobacter salinestris* YRNF3 and eight different species from genus *Azotobacter* from the GenBank database. Bootstrap values are shown on the nodes. The scale bar represents the number of nucleotide substitutions per site.

The diazotrophic bacteria YRNF3 was molecularly identified based on its 16S rDNA region. The result indicated that the amplified nucleotide sequence (1393 bp) of the diazotrophic bacteria YRNF3 had 99.78% similarity with *A. salinestris* (CP045302). Based on this result, the diazotrophic bacteria YRNF3 was identified as *A. salinestris* YRNF3 and its nucleotide sequence was deposited in the GenBank database under the accession number (OQ605418). Phylogenetic analysis of *A. salinestris* YRNF3 with different species in genus *Azotobacter* from the GenBank database revealed that all species were clustered into two distinct clads. One of them contained two species *A. beijerinckii* (FNYO01000222) and *A. chroococcum* (SWKB01000103) with a 86% bootstrap support, while the other species were clustered in the other clade under two subclades, one of them contained *A. salinestris* YRNF3 (OQ605418), *A. salinestris* (JN641802) and *A. vinelandii* (OQ415270) with a bootstrap support (92%). In the other subclade, four species were grouped, namely *A. tropicalis* (MW586885), *A. nigricans* (LN874291), *A. armeniacus* (OP978160), and *A. bryophylli* (MF078077) with a 88% bootstrap support (Fig. 3).

### Estimation of the calcite-solubilization potential under in-vitro conditions

Calcite-solubilization potential of *A. salinestris* YRNF3 was estimated along 10 days of incubation under in-vitro conditions as follows:

#### pH

**Figure 4:**
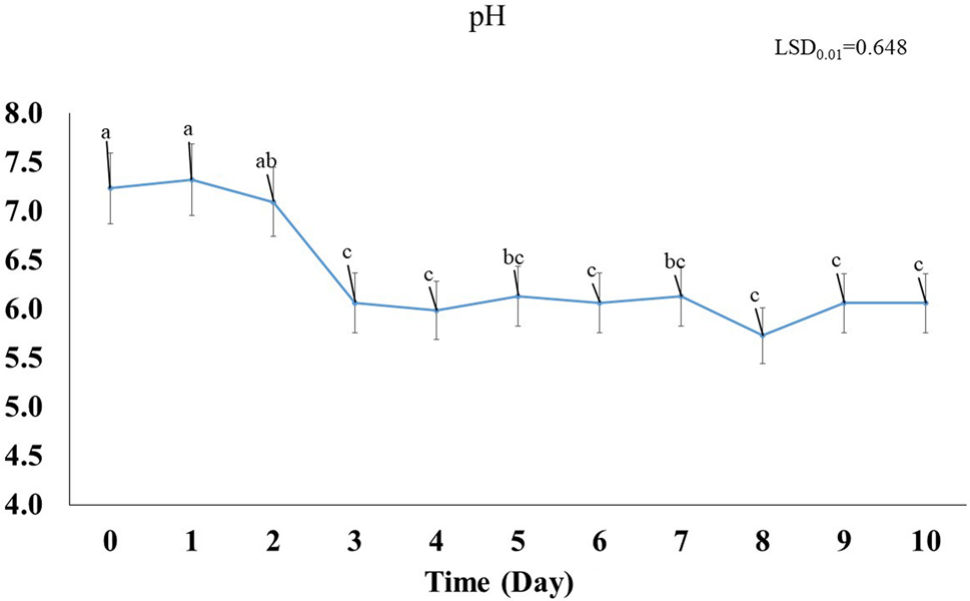
Variation in pH values of the culture filtrate of *Azotobacter salinestris* YRNF3 along 10 days of incubation. Values superscripted with the same letter are not significantly different according to Tukey’s HSD test at *p* ≤ 0.01. Each value represents the mean of three replicates. Error bars represent standard errors. LSD = least significant difference.

The pH value ranged from 5.73 to 7.32, compared with the initial pH 7.24 (Fig. 4). The pH significantly (*p* < 0.01) fluctuated throughout the experiment, reaching a minimum of 5.98 on the day 4, compared to the control (day 0). The pH then increased gradually until day 7, followed by a slight decrease on day 8 (Fig. 1). As shown in Fig. 4, application of *A. salinestris* YRNF3 led to a lowering in the pH of the culture filtrate with the increment in the incubation period. The pH value at day 8 recorded the lowest value, while no significant differences (*p* < 0.01) were observed among days 3, 4, 6, and 8–10. The pH at days 0–2 showed the highest values. No significant difference was observed between the pH value at days 5 and 7.

The observed changes in pH values can be attributed to several factors such as changes in the dissolved CO_2_ level, production of acidic or alkaline metabolites, or to the bacterial activity^2,3,31^. However, the pH values observed in this study were within the acceptable range for the bacterial growth, which is typically ranged between 6.5 and 8.5. During the bacterial growth, various acidic or alkaline metabolites can be produced and modulate the pH of the culture^32^. In particular, production of ammonia and organic acids, in this case, as noticed in the next results. In addition, level of the dissolved CO_2_ in the culture can also affect the pH value, as the dissolved CO_2_ can react with water to form carbonic acid, which can lower the pH value of the culture. In the same time, the pH value of the soil may affect the type and quantity of the bacterial population that can grow in this soil^32^. Reaction of the dissolved CO_2_ and water to form carbonic acid proceeds according to the following equation:

$${\text{CO}}_{{2}} + {\text{ H}}_{{2}} {\text{O}} \rightleftharpoons {\text{H}}_{{2}} {\text{CO}}_{{3}}$$

This reaction can affect the pH of the culture as the carbonic acid can donate H^+^ ions, leading to a decrease in the pH^33,34^. While solubilization of calcium carbonate in water proceeds as follows:

$${\text{CaCO}}_{{3}} + {\text{ H}}_{{2}} {\text{O }} + {\text{ CO}}_{{2}} \rightleftharpoons {\text{Ca}}^{{{2} + }} + {\text{ 2HCO}}_{{3}}$$

This reaction results in an increment in the concentration of the bicarbonate ion leading to a decrease in the pH. On the other hand, the calcareous soils typically have a high pH due to the high content of calcium carbonate. This pH increment can affect the type and population of the inhabitant microorganisms^2,3,31^. In this case, addition of acids-producing bacteria can potentially help in lowering the pH to a more neutral range. This may create a more favorable environment for the growth of various microorganisms as well as increase the nutrients availability to the growing plants. However, it is important to note that the specific effects of the inhabiting bacteria on the calcareous soil will depend on other factors such as the composition of the soil and the type of the present microorganisms^10^.

Furthermore, the existing microorganisms in the soil, including both bacteria and other microbial communities, contribute to the overall soil ecosystem. Interactions between different microorganisms can be cooperative or competitive, and they can have synergistic or antagonistic effects on each other. The presence of specific microorganisms can influence the activities and functions of bacteria in the soil, including nutrient cycling, organic matter decomposition, and disease suppression^10^.

In summary, while the presence of bacteria in the calcareous soil can has effects on the soil processes, the specific outcomes will be influenced by other factors such as soil composition, nutrient availability, pH, and the interactions with other microorganisms. Understanding these complex interactions is crucial for comprehending the overall dynamics of the soil ecosystem and its implications for the plant growth, nutrient cycling, and soil health^32^.

#### Calcium concentration

**Figure 5:**
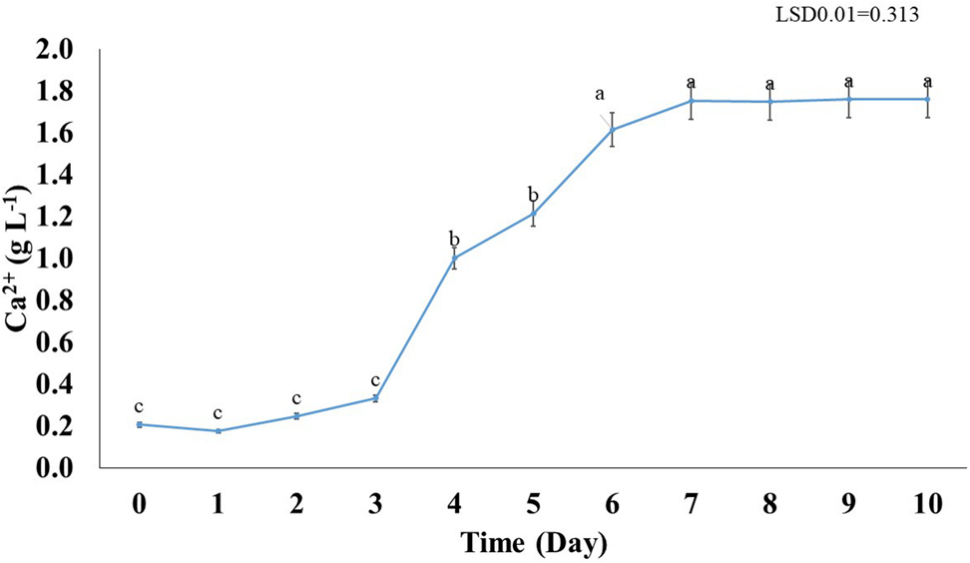
Gradual increment in the calcium concentration in the culture of *Azotobacter salinestris* YRNF3 along the time of the experiment. Values superscripted with the same letter are not significantly different according to Tukey’s HSD test at *p* ≤ 0.01. Each value represents the mean of three replicates. Error bars represent standard errors. LSD = least significant difference.

Calcium concentration ranged from 0.17 to 1.76 gL^−1^. The initial calcium concentration in the bacterial culture on day 0 was 0.21 gL^−1^, and the highest concentration was observed on day 10 recording 1.76 gL^−1^ (Fig. 5).

Figure 5 illustrates the impact of *A. salinestris* YRNF3 on the calcium carbonate solubilization in the culture filtrate. Results showed that culturing of *A. salinestris* YRNF3 led to a significant increment (*p* < 0.01) in the calcium concentration in the culture medium from day 4 to 10, compared to the control. No significant difference was observed in the calcium concentration among days 1–3. However, the calcium concentration at days 6–10 had the highest values without any significant difference (*p* < 0.01) between them.

The fluctuation in the calcium concentration can be attributed to the solubilization of calcium-bearing minerals and cellular metabolic processes^10^. The observed fluctuation in calcium concentration along the studied period suggests that calcium is a dynamic and complex nutrient. The initial calcium concentration on day 0 was relatively low. However, as the experiment progressed, the calcium concentration gradually increased, reaching the maximum on day 10. This increment in the calcium concentration indicates the calcite-solubilization by *A. salinestris* YRNF3.

On the other hand, the observed fluctuations in the calcium concentration can provide significant implications for the calcareous soil reclamation. Calcareous soils are characterized by a high content of calcium carbonate, which causes soil alkalinity and reduces the availability of certain nutrients to the plants^2^. Depending on the solubility product of CaCO_3_, this solubilization results in a high HCO_3_^−^ concentration that buffers the soil pH in the range of 7.5 to 8.5 which can enhance the nutrients availability in the soil^34^. Therefore, addition of CSB can potentially enhance the soil fertility and promote the plant growth^13^. Modulation of the bicarbonate ion in the soil by calcium is achieved according to the following equation:

$${\text{Ca}}^{{{2} + }} + {\text{ 2HCO}}_{{3}}^{ - } \rightleftharpoons {\text{Ca }}\left( {{\text{HCO}}_{{3}} } \right)_{{2}}$$

Calcium is a crucial nutrient for maintaining of the healthy plants and fertile soils, influencing vital physiological processes such as cell division, nutrient uptake, and cell wall development, in addition to its contribution into the properties of soil aggregates^35,36^.

#### Bicarbonate concentration

**Figure 6:**
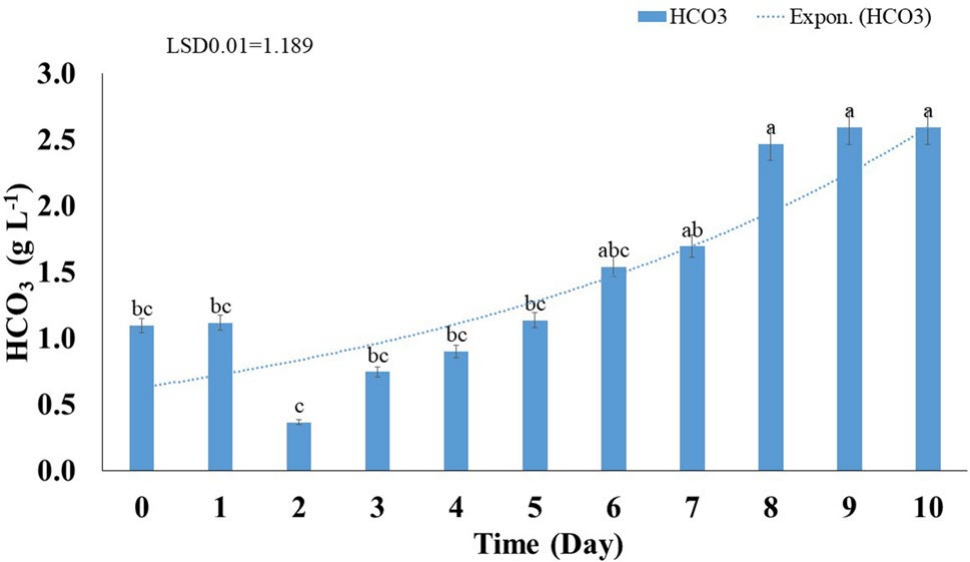
Fluctuation of the bicarbonate concentration in the culture of *Azotobacter salinestris* YRNF3 along the time of the experiment. Values superscripted with the same letter are not significantly different according to Tukey’s HSD test at *p* ≤ 0.01. Each value represents the mean of three replicates. Error bars represent standard errors. LSD = least significant difference.

The obtained results revealed that the bicarbonate concentration in the culture medium ranged between 0.37 and 2.59 gL^−1^. The highest concentration (2.59 gL^−1^) was observed on day 10. The initial bicarbonate concentration on day 0 was 1.09 gL^−1^ (Fig. 6). The bicarbonate concentration showed the highest values on days 8–10 compered to days 0–7, without any significant difference (*p* < 0.01) between them. While it had the lowest value in days 0–5 without any significant difference between them at (*p* < 0.01). Bicarbonate is an important buffer system that regulates pH levels in water bodies^34^. Bicarbonate content can neutralize the soil pH, as illustrated in the following equation:

$${\text{HCO}}_{{3}}^{ - } + {\text{ H}}^{ + } \to {\text{ CO}}_{{2}} + {\text{ H}}_{{2}} {\text{O}}.$$

This chemical reaction generates carbon dioxide (CO_2_) and water (H_2_O), which provide plants with a source of carbon., Bicarbonate-based amendments can enhance the overall quality of the calcareous soil by balancing the soil pH and providing carbon for the plant growth. However, the specific effects of bicarbonate addition to the calcareous soil depend on various factors such as the composition of the soil and the type of the grown plants. The obtained results in this study suggested that application of *A. salinestris* YRNF3 may be effective in the calcareous soil reclamation. By adding the organic acids, the soil pH can be decreased leading to an improvement of the nutrients availability for the grown plants. In addition, incorporating the diazotrophic bacteria to the soil can be a viable solution for improving the quality of calcareous soil^33,37^.

#### The total nitrogen content

The total nitrogen (TN) content encompasses various forms of nitrogen, including nitrate (NO_3_^−^), nitrite (NO_2_^−^), ammonia (NH_3_), ammonium (NH_4_^+^), and organic nitrogen. This relationship can be expressed by the following equation^20,21^

$${\text{TN }} = {\text{ NO}}_{{3}}^{ - } + {\text{ NO}}_{{2}}^{ - } + {\text{ NH}}_{{3}} + {\text{ NH}}_{{4}}^{ + } + {\text{ organic nitrogen}}$$

**Figure 7:**
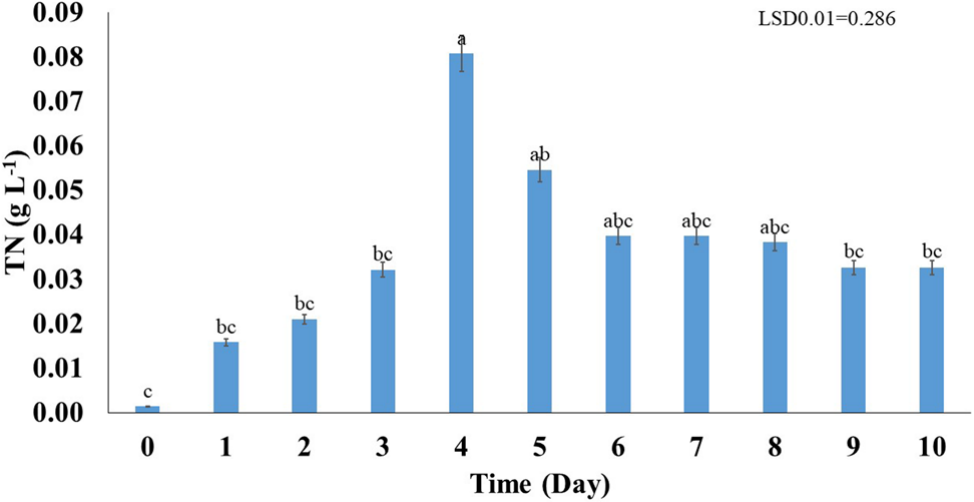
Fluctuation of total nitrogen (TN) concentration in the culture of *Azotobacter salinestris* YRNF3 along the time of the experiment over time. Values superscripted with the same letter are not significantly different according to Tukey’s HSD test at *p* ≤ 0.01. Each value represents the mean of three replicates. Error bars represent standard errors. LSD = least significant difference.

Results obtained indicated that the TN content in the bacterial culture ranged from 0.0014 to 0.0807 gL^−1^. The highest concentration was observed with a high significance (*p* < 0.01) on days 4 and 5, compared with the other days (Fig. 7). On other hand, the TN content in day 0 had the lowest value, compared to the all days. The incubation time from days 1–3, 9,10 and 6–8 showed no significant differences between them, compared with days 4 and 5. This confirms the nitrogen-fixing ability of *A. salinestris* YRNF3. Conversion of the atmospheric nitrogen (N_2_) into ammonia (NH_3_) is known as the biological process of nitrogen fixation. This process is carried out by certain microorganisms that called diazotrophic bacteria such as *A. salinestris* YRNF3 and can be represented by the following equation^38^

$${\text{N}}_{{2}} + {\text{ 8H}}^{ + } + {\text{ 8e}}^{ - } + {\text{ 16 ATP }} \to {\text{ 2NH}}_{{3}} + {\text{ H}}_{{2}} + {\text{ 16ADP }} + {\text{ 16Pi}}$$

Nitrogen fixation is a critical reaction in the nitrogen cycling and is essential for the growth and survival of microorganisms and plants^39^. However, it is important to note that the effects of soil nitrogen levels on the bacterial growth depend on various factors, such as the composition of the soil and the type of microorganisms present. However, the excessive nitrogen content in the bacterial culture can lead to downregulation of the nitrogenase gene resulting in the suppression of the nitrogen fixation process as follows^16^:

$${\text{NifA }} + {\text{ 2ATP }} + {\text{ 2e}} - \, + {\text{ NifL }} \to {\text{ NifA}}* \, \to {\text{ reduced nitrogenase gene expression}}$$

Furthermore, application of the diazotrophic bacteria for soil improvement should be carefully managed to avoid excessive nitrogen accumulation and the associated negative effects on the soil and water quality^13,20^.

#### The total protein content

**Figure 8:**
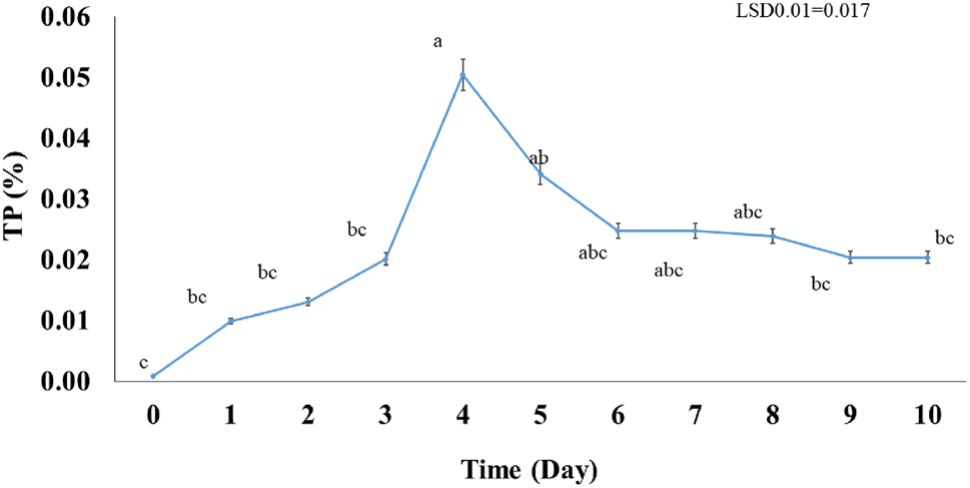
Total protein content in the culture of *Azotobacter salinestris* YRNF3 along the time of the experiment. Values superscripted with the same letter are not significantly different according to Tukey’s HSD test at *p* ≤ 0.01. Each value represents the mean of three replicates. Error bars represent standard errors. LSD = least significant difference.

The total protein (TP) content produced by *A. salinestris* YRNF3 along the ten days of incubation ranged between 0.0009 and 0.0505% (Fig. 8). The highest content was recorded in the day 4 (0.0505%), while no TP content was recorded on the day 0. From the data presented in Fig. 8, we can see that the TP content reached its highest value on the day 4. After which its value gradually decreased from day 5 to 8. While the TP content values were constant on days 9–10 without any significant difference at (*p* < 0.01) between them.

Proteins are essential for the bacterial growth and playing an important role in many physiological processes including the growth and development. The observed fluctuation in the TP can be attributed to several factors such as the nitrogen fixation rate, nutrient uptake by the bacteria, and the release of proteins from the dead cells. The increment in TP in day 4 can be attributed to the growth and replication of bacteria that utilize proteins as a nutrient source. As the bacterial growth and reproduction increase, more proteins are consumed, leading to a decrease in the TP. Proteins can be synthesized from nitrogen-containing amino acids, which can be obtained from a variety of sources, including nitrogen gas (N_2_) in the air. Ammonia (NH_3_) produced by the nitrogen fixation can be assimilated into amino acids and other nitrogen-containing compounds as follows^40^:

$${\text{Amino acids }} + {\text{ Ribosomes }} + {\text{ ATP }} + {\text{ tRNAs }} \to {\text{ Protein }} + {\text{ Water }} + {\text{ AMP }} + {\text{ PPi }} + {\text{ Release factors}}$$

Synthesis of proteins from nitrogen is a critical process in the growth and development of living organisms, and the ability to fix atmospheric nitrogen provides an important source of nitrogen for this process^16^.

### Quantification of the organic acids produced by *A. salinestris* YRNF3

**Figure 9:**
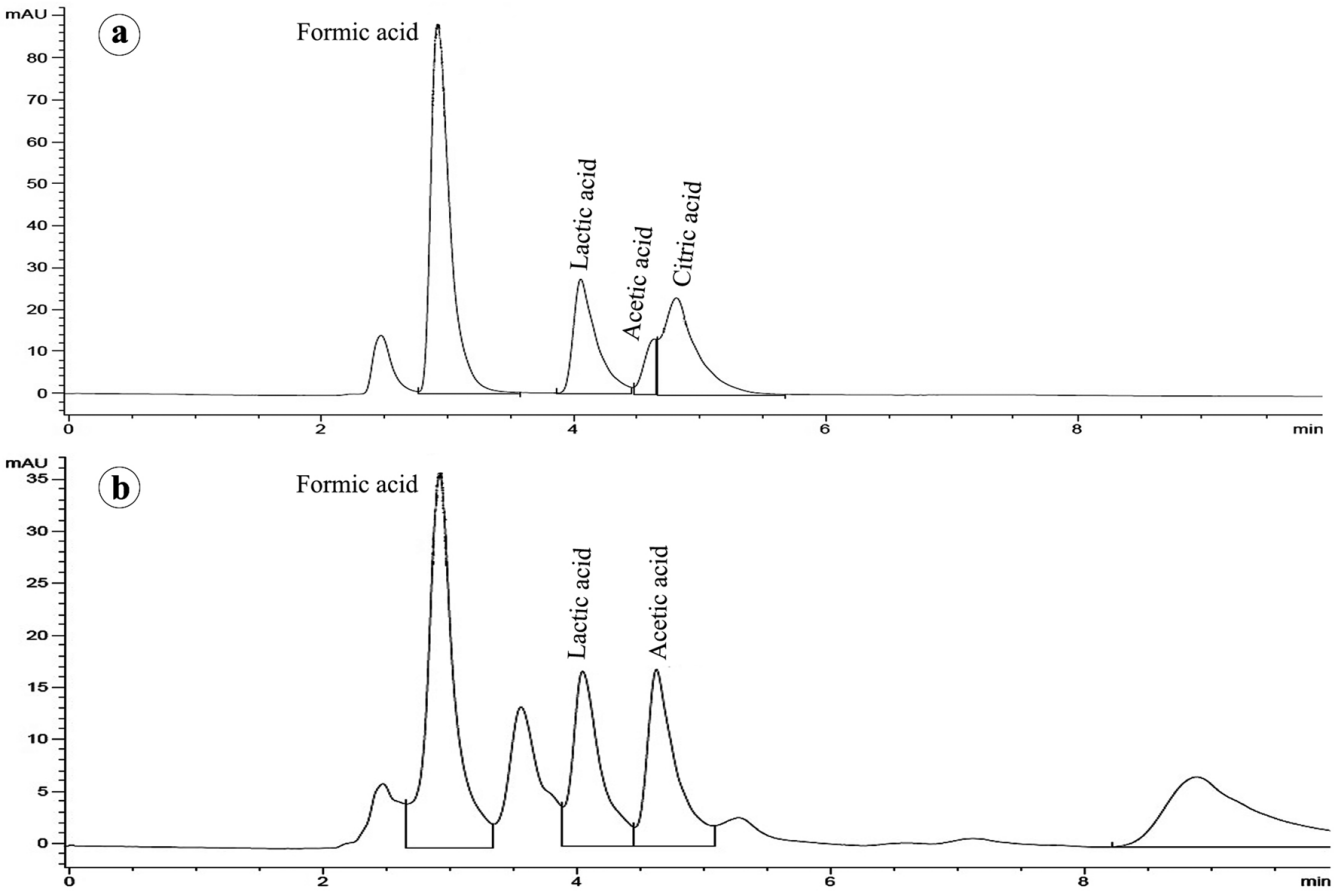
A HPLC chromatogram shows the organic acids produced by *A. salinestris* YRNF3, where (**a**) a mixture standard solution containing formic, lactic, acetic, and citric acids, and (**b**) the culture filtrate of *A. salinestris* YRNF3.

Results obtained from the HPLC analysis revealed that *A. salinestris* YRNF3 produced three organic acids in its culture filtrate, at varying extents, including formic, lactic, and acetic acids (Fig. 9, and Table 2). The major produced organic acid was lactic acid (82.57 mg mL^−1^), followed by formic acid (46.8 mg mL^−1^), while acetic acid was detected in a low quantity (3.11 mg mL^−1^). Based on this result, the calcite-solubilization ability of *A. salinestris* YRNF3 can be attributed mainly to the produced lactic acid. Solubilization of calcium carbonate by acidification has been widely studied in the last years^41,42^. Acidic solubilization of calcite is proceeded according to the following equation:

$${\text{H}}^{ + } + {\text{ CaCO}}_{{3}} \rightleftharpoons {\text{Ca}}^{{{2} + }} + {\text{ HCO}}_{{3}}^{ - }$$

**Table 2:** A HPLC profile of some organic acids produced by *A. salinestris* YRNF3.

Name	Retention time (min)	Concentration (mg mL^−1^)
Formic acid	2.922	46.80
Lactic acid	4.049	82.57
Acetic acid	4.631	3.11
Citric acid	4.822	–

Gray et al. (2018) found that the solubilization of calcite by hydrochloric acid (HCl) in porous media is primarily controlled by the transport of acid into the media, with the increment in the solubilization rate the acid concentration increases and the pore size decreases. The authors also observed the formation of etch pits and channels on the surface of the calcite crystals, suggesting that the solubilization process is selective and influenced by the crystallographic orientation of the calcite. The study provides valuable insights into the chemical mechanisms involved in the solubilization of calcite in the porous media, with important implications for understanding the geochemical processes in the natural and engineered systems.

The solubilization of calcite by hydrochloric acid (HCl) in porous media can be represented by the following chemical equation:

$${\text{CaCO}}_{{3}} + {\text{ 2HCl }} \to {\text{ CaCl}}_{{2}} + {\text{ CO}}_{{2}} + {\text{ H}}_{{2}} {\text{O}}$$

Organic acids are characterized with low corrosivity and slow reaction rates, which qualify them for the calcite solubilization. However, lactic acid has a significant advantage over other organic acids, which is its high solubility at high temperatures, forming calcium lactate^43^. Formic acid has the same strength of lactic acid, and a dissociation constant higher ten times than the acetic acid^44^.

Al-Khaldi et al.^40^ described a study that investigated the reaction of citric acid with calcite. The authors used a variety of experimental techniques to measure the reaction kinetics and mass transfer of citric acid with calcite under different conditions such as temperature, pH, and concentration. The results of the study showed that the reaction rate of citric acid with calcite is influenced by several factors, including the mineral surface area, the concentration of citric acid, and the pH of the solution. The reaction of citric acid with calcite can be represented by the following chemical equation:

$${\text{3H}}_{{3}} {\text{C}}_{{6}} {\text{H}}_{{5}} {\text{O}}_{{7}} + {\text{ 4CaCO}}_{{3}} \to {\text{ 4CO}}_{{2}} + {\text{ 3H}}_{{2}} {\text{O }} + {\text{ 2Ca}}_{{3}} \left( {{\text{C}}_{{6}} {\text{H}}_{{5}} {\text{O}}_{{7}} } \right)_{{2}}$$

The production of the organic acids by *A. salinestris* YRNF3 is likely a survival mechanism for the bacterium in its natural environment. Organic acids can act as chelators, which are molecules that bind to metal ions and increase their solubility^41,42^. By producing organic acids such as lactic acid, *A. salinestris* YRNF3 may be able to access and utilize nutrients such as calcium, which are typically not readily available in its environment. In addition, the production of organic acids may help *A. salinestris* YRNF3 to compete with other microorganisms by lowering the pH in its environment, which can inhibit the growth of other bacteria^20,21^.

Furthermore, the specific organic acids produced by *A. salinestris* YRNF3 may be influenced by various environmental factors such as temperature, pH, and nutrient availability. For instance, lactic acid production may be favored at higher temperatures due to its high solubility, while formic acid may be produced in response to low nutrient availability^44,57^.

Rabie et al.^41^ found that the reaction rate of lactic acid with calcite and dolomite is influenced by several factors, including the mineral surface area, the concentration of lactic acid, and the pH of the solution. The study showed that the reaction rate increased with increasing temperature and decreasing pH, and the calcite was more reactive than dolomite under the same conditions. The results have important implications for understanding the geochemical processes that occur in subsurface environments, and may have practical applications in areas such as acidizing and hydraulic fracturing of carbonate reservoirs^41,42^.

The reaction of lactic acid with calcite and dolomite can be represented by the following chemical equation:

$${\text{CaCO}}_{{3}} + {\text{ 2CH}}_{{3}} {\text{CH}}\left( {{\text{OH}}} \right){\text{COOH }} \to {\text{ Ca }}\left( {{\text{CH}}_{{3}} {\text{CH}}\left( {{\text{OH}}} \right){\text{COO}}} \right)_{{2}} + {\text{ CO}}_{{2}} + {\text{ H}}_{{2}} {\text{O}}$$

Overall, the study provides valuable insights into the reaction kinetics and mass transfer of lactic acid with calcite and dolomite, which may help to improve our understanding of carbonate solubilization mechanisms and their effects on subsurface fluid flow and transport.

### Activity of acid phosphatase produced by *A. salinestris* YRNF3

Data obtained revealed that *A. salinestris* YRNF3 produced acid phosphatase at low activity (5.4 U.mL^−1^). This result can be discussed in the light of the quantity and form of the phosphorus content in the culturing medium, where it exists in a low quantity and in the form of orthophosphate, which is readily available for the microbial uptake. In this case, the bacteria are not in need to the activity of phosphatase. Phosphorus is a vital nutrient for plants and microorganisms and involved in many physiological processes such as energy transfer, metabolism, and membrane transport. Under soil conditions, the high weathering rate and the geochemical binding of phosphorus to iron and aluminum result in an orthophosphate devoid soil^45^. In order to alleviate the phosphorus limitation, some of the soil microorganisms such as arbuscular mycorrhizal fungi and phosphorus-dissolving bacteria are important to regulate the phosphorus acquisition by production of acid and alkaline phosphatases^46^. However, phosphorus availability is a highly dependent on many factors such as soil pH, sorption–desorption and solubilization–precipitation equilibriums. Acid and alkaline phosphatases can solubilize phosphorus from these minerals^47^.

### Conclusions

*Azotobacter salinestris* YRNF3 was found to has a calcite-solubilization potential. Based on the obtained results, it can be concluded that *A. salinestris* YRNF3 is a promising candidate for application in reclamation of the calcareous soils. However, further studies are required to evaluate application of *A. salinestris* YRNF3 in the open large-scale field. The findings of this work provide insights into the calcite-solubilization property of the diazotrophic *A. salinestris* YRNF3 and contribute to understand the role of biological systems in CaCO_3_ solubilization. To the best of the author's knowledge, this is the first study dealing with the calcite-solubilization ability of a diazotrophic bacteria.

## Materials and methods

### Soil samples

Soil samples were collected from different agricultural fields in different Egyptian governorates representing variable climatic conditions and soil types, namely Alexandria, Al-Behera, New Valley, Port Said, and Sinai during 2021. The sampling sites were georeferenced using the global positioning system (GPS) to ensure accurate location data. For each site, 250 g of soil was carefully collected using a sterile spatula at a depth of 10 cm. The sampling process aimed to obtain representative soil samples from the rhizosphere, the region of soil surrounding plant roots where active nutrient exchange and microbial activity occur. The agricultural fields, from which the soil samples were taken, were cultivated indicating that they were actively used for agricultural purposes. The specific crop grown in these cultivated soils was wheat.

To maintain the integrity and quality of the soil samples, they were collected in sterile plastic bags, labeled in the field, and immediately transferred to the laboratory. The samples were stored at 4 °C until further analysis and investigation^48^.

### Isolation of the diazotrophic bacteria

Ten grams of each soil sample were suspended in 90 mL sterile water and shaken at 120 rpm using a rotary at room temperature for 15 min. The suspension was then serially diluted and 1 mL was spread onto NF medium and incubated at 30 °C for 5 days. The used medium composed of mannitol (20 gL^−1^), CaCO_3_ (5 gL^−1^), NaCl (0.2 gL^−1^), K_2_HPO_4_ (0.2 gL^−1^), K_2_SO_4_ (0.1 gL^−1^), MgSO_4._7H_2_O (0.2 gL^−1^), and agar (20 gL^−1^) (Merck KGaA, Darmstadt, Germany). The pH of the medium was adjusted at 6.5. After the incubation period, colonies of the grown bacteria were picked according to the shape, color, and size of the colony and purified before storing in glycerol (20%) at − 20 °C until use^49^.

### Ammonia production test

All isolated bacteria were qualitatively tested for ammonia production using Nessler’s reagent. Each bacterial isolate was inoculated in a sterilized glass tube containing 4% peptone broth and incubated for a week at 30 °C. After the incubation period, 0.5 ml of Nessler’s reagent was added to each tube. Ammonia production was positively detected by the development of a brown to yellow color^50^.

### Amplification of nitrogenase gene

Total DNA of the bacterial isolate was extracted using a QiAamp DNA Mini Kit (Qiagen, Hilden, Germany). To amplify the nitrogenase gene (*nifH*), the primers *nifH*-F 5’-AAAGGYGGWATCGGYAARTCCACCAC-3’ and *nifH*-R 5’-TTGTTSGCSGCRTACATSGCCATCAT-3’ were used. The PCR reaction mixture (25µL) composed of 5 × reaction buffer (10 µL), dNTPs (2 mmol L^−1^, 5 µL), primers F and R (10 µM, 1 µL for each primer), DNA (2 µL), DNA polemerase (2 U µL^−1^, 0.25 µL), and dd H_2_O (8.75 µL). The PCR program was performed as follows: 5 min at 95 °C, 40 cycles (5 s at 95 °C, 30 s at 57 °C, and 40 s at 72 °C). The amplified product was electrophoresed on an agarose gel and imaged using a gel documentation system^51^.

### Screening of the isolated bacteria for calcite-solubilizing activity

The isolated bacteria were screened for their calcite-solubilization activity. Each bacteria was cultured on DBA medium which consisted of glucose (5 gL^−1^), yeast extract (1 gL^−1^), peptone (1 gL^−1^), CaCO_3_ (5 gL^−1^), NaCl (5 gL^−1^), K_2_HPO_4_ (0.4 gL^−1^), (NH_4_)_2_SO_4_ (0.05 gL^−1^), MgSO_4_ (0.01 gL^−1^), and agar (20 gL^−1^)^52^. The positive isolate was selected based on the clear zone formation around the colony. The solubilization index of an isolate was determined by measuring diameters of the clear zone and the colony size as follows:

$$\mathrm{Solubilization\, index }= \frac{\mathrm{Clear\, zone }+\mathrm{ Colony\, size }}{\mathrm{Colony\, size}}$$

### Molecular identification of the selected isolate

Total DNA of the selected isolate based on its calcite-solubilization potential using the QiAamp DNA Mini Kit (Qiagen, Hilden, Germany). Amplification of the 16S rDNA region was performed using the primers 16S-27F 5’-AGAGTTTGATCMTGGCTCAG-3’ and 16S-1492R 5’-CGGTTACCTTGTTACGACTT-3’ as described by White et al.^53^. Sequence of the amplified product was aligned and compared to the GenBank database via BLAST. Phylogeny tree of the selected isolate compared to the closest sequences from the GenBank database was generated using the maximum likelihood method using MEGA X software (10.2.4).

### Estimation of the calcite-solubilization potential under the in-vitro conditions

Calcite-solubilization potential of the selected diazotroph YRNF3 was estimated along 10 days of incubation under the in-vitro conditions. The bacterial isolate was cultured on NF broth (see 2.2.) and incubated under shaking at 120 rpm at 30 °C for 10 days. At each day, the bacterial culture was centrifuged at 3000 rpm for 15 min and the cell free supernatant was analyzed for the soluble calcium^54^, bicarbonate^55^, total nitrogen by Kjeldahl method^56^, total protein using Bradford reagent^57^, and pH using a pH meter. All analyses were performed in triplicates.

### High-performance liquid chromatography (HPLC)

A cell-free culture filtrate of the diazotrophic bacteria YRNF3 was assessed for the organic acids production using a HPLC system (Agilent Technologies, Santa Clara, CA, USA) with a UV detector. The chromatographic separation was done using a reversed-phase column (Extend-C18, column, 4.6 mm × 250 mm, 5 μm particle size) with acetonitrile in H_2_SO_4_ as a mobile phase. The mixture standard solution (formic acid, lactic acid, acetic acid, and citric acid) was used for the organic acids detection^10^.

### Quantification of the acid phosphatase activity

The cell-free supernatant of the diazotrophic bacteria YRNF3 was used as a crude enzyme. Activity of the acid phosphatase was estimated as described by Abdelgalil et al.^58^. The reaction mixture, composed of the crude enzyme, 100 mM sodium acetate buffer pH 4, and 1 mM *ρ-*nitrophenylphosphate, was incubated at 65 °C. The color was measured using a UV–visible spectrophotometer at 405nm.

#### Statistical analyses

The data obtained were analyzed using the statistical software CoStat (version 6.4). One-way analysis of variance (ANOVA) was used to test for significant differences between the means of the experimental groups. Tukey's HSD test at *p* ≤ 0.05 was used for the post-hoc analysis to determine the differences between the individual groups.

## Data Availability

Data that support these results are available from the corresponding author upon request. Nucleotide sequence of *A. salinestris* YRNF3 was deposited in the GenBank database under the accession number (OQ605418).
